# Terahertz time-domain spectroscopy of edible oils

**DOI:** 10.1098/rsos.170275

**Published:** 2017-06-28

**Authors:** Alex Dinovitser, Dimitar G. Valchev, Derek Abbott

**Affiliations:** 1 School of Electrical and Electronic Engineering, University of Adelaide, Adelaide, South Australia, 5005, Australia; 2 School of Engineering and Innovation, The Open University, Milton Keynes MK7 6AA, UK

**Keywords:** terahertz, photonics, food science, edible oils

## Abstract

Chemical degradation of edible oils has been studied using conventional spectroscopic methods spanning the spectrum from ultraviolet to mid-IR. However, the possibility of morphological changes of oil molecules that can be detected at terahertz frequencies is beginning to receive some attention. Furthermore, the rapidly decreasing cost of this technology and its capability for convenient, *in situ* measurement of material properties, raises the possibility of monitoring oil during cooking and processing at production facilities, and more generally within the food industry. In this paper, we test the hypothesis that oil undergoes chemical and physical changes when heated above the smoke point, which can be detected in the 0.05–2 THz spectral range, measured using the conventional terahertz time-domain spectroscopy technique. The measurements demonstrate a null result in that there is no significant change in the spectra of terahertz optical parameters after heating above the smoke point for 5 min.

## 1. Introduction

Edible cooking oils form a major part of food production and human nutrition. From frying through baking to salad dressing, they are practically omnipresent in every cuisine. However, there may be serious health implications due to undesirable constituents in fats and oils [1], and how their composition changes with cooking and processing. Cooking oils are known to undergo physical changes including distillation (evaporation of volatiles), dehydration and polymerization [2]. Additionally, chemical reactions include hydrolysation of triacylglycerols as well as oxidation to hydroperoxides [2].

Previous studies have revealed spectral features in edible oils at terahertz frequencies [3–5], as well as significant differences in the terahertz spectra of oils that have undergone extensive processing in contact with food [6] as well as the reprocessing and recycling of used oils [7]. One of the main uses for edible oils in industry is frying, where the oil may boil at or above its smoke point and the thermal processing affects the molecular structure of the long chains of carbon and hydrogen atoms comprising the fatty acids. After cooling down, the molecular structure of the edible oil is different to that of its raw state [8]. Note that no prior studies have been performed in the terahertz range that take edible oils to their smoke point temperature.

The large production and distribution volume of edible oils, coupled with health considerations associated with them, require a unified objective quality control methodology and the development of fast non-contact and non-destructive methods for identification and quantitative characterization of the composition and structure of substances in edible oils. Therefore, a method for the rapid, reliable and low-cost *in situ* characterization of edible oils is a challenging but important goal.

A number of techniques exist to characterize and measure the health, safety and nutritional properties of edible oils. These can be broadly classified into five categories as follows [9,10]:

(i) chemical methods employing reagents, and sometimes optical instruments to measure colour changes that characterize properties including acidity, peroxide, iodine value, saponification, etc.,
(ii) physical separation methods employing various chromatographic and related techniques,
(iii) nuclear magnetic resonance (NMR) spectroscopy,
(iv) mass spectrometry, and
(v) various electromagnetic spectroscopic techniques, including terahertz spectroscopy.

While chemical and physical chromatographic [11] techniques can yield comprehensive quantitative measurements of structure and composition, these techniques require numerous preparation steps, are slow and laborious, and generally cannot be performed *in situ* [12].

NMR and pyrolytic mass spectrometry primarily yield information about the elemental composition [13], from which the overall composition such as the carbon–hydrogen ratio polysaturation can be derived [14]. However, the detailed molecular composition and structure of greatest relevance to human health can only sometimes be indirectly inferred using sophisticated analysis techniques [15], often requiring additional chromatographic or chemical assays [16]. Therefore, these techniques have not seen much industrial applicability to date.

Electromagnetic spectroscopic techniques, on the other hand, receive the greatest level of interest because of the potential for low-cost, rapid and *in situ* measurement. Each molecular functional group has a unique spectrum over some frequency band, where each resonance line in the spectrum corresponds to a quantized vibrational-rotational mode of a dipole interaction. The pattern of the precise centre frequencies and relative magnitudes of the resonances can serve as a fingerprint for the purpose of identification. These techniques have been employed for oil analysis over a wide spectrum from ultraviolet to terahertz, and broadly speaking, measure the interactions between the electric field and molecular dipoles, with high resolution. Chemical changes and degradation in edible oils have been characterized using ultraviolet (UV), visible light (Vis) [17], mid-infrared (MIR) and near-IR (NIR) spectroscopic techniques [18], as well as Raman spectroscopy [19]. Measurements at the longer mid-IR wavelengths revealed further spectral details, however, high attenuation in this spectral band requires the use of the attenuated total reflection (ATR) technique, as well as the use of specialized window materials [20]. Raman techniques can provide similar spectra as that available at longer wavelengths, however, these measurements still require transmission measurements at shorter wavelengths, as well as fine spectral discrimination.

As cooking oils are known to undergo oxidative modification when exposed to the oxygen of air at elevated temperatures, there has been considerable success recently in the identification of heat-damaged oils using infrared spectroscopy [6,7]. These studies use Fourier transform IR (FTIR), and reveal spectral detail at longer wavelengths. However, these studies are typically in the mid-infrared portion of the spectrum that is limited to optical wavenumbers above 500 cm^−1^, or wavelengths shorter than 20 μm (greater than 15 THz).

Terahertz waves carry frequencies in the range between the microwave and the infrared bands, and lie between 0.1 and 10 THz [21]. These share properties of both radio waves and visible light and interact with molecular structures on a larger scale where dipoles are more distant and weakly bound with a larger mass. These structures have resonances at lower electromagnetic frequencies or longer wavelengths, spanning the far-infrared (FIR) and terahertz (THz) bands. Spectral measurements in these bands have potential to reveal the structural dynamics of substances at the intramolecular level through hydrogen bonds, and at the intermolecular level through van der Waals forces [3]. Crystalline phonon vibrations, hydrogen bond stretching and molecular torsion mode frequencies commonly occur in the terahertz range [22], and the terahertz resonances at the corresponding frequencies for the substance under investigation, serve as a signature or fingerprint. Spectral features at FIR and terahertz frequencies are, therefore, of interest because they may reveal molecular structural changes and molecular morphology [23,24] that cannot be detected at shorter wavelengths [25].

Furthermore, terahertz technology is relatively new and rapidly evolving, with the prospect of low-cost application with desirable practical advantages. Terahertz measurements are non-destructive and non-invasive. Like radio waves, terahertz waves can penetrate many packaging materials such as plastics and cardboard. Like visible light, terahertz waves can be reflected and focused using conveniently sized mirrors and lenses. Furthermore, terahertz waves have an overall low level of absorption by fat and, therefore, the vibration modes of fatty acids can be analysed in a simple transmission setting with reasonably long path lengths that do not require techniques such as ATR [26]. Furthermore, the thickness of the oil sample can be tailored to achieve the desired level of attenuation, that eliminates the need to for specialized techniques such as differential spectroscopy.

Unlike shorter-wavelength radiation that requires clean optical windows for efficient transmission, terahertz waves easily pass through materials such as PTFE that are naturally resistant to fouling, as may be encountered at a food processing facility, which makes this terahertz technology potentially particularly attractive for quality control.

The purpose of this work is therefore to extend the study of the resonance spectra of common edible oils into the terahertz portion of the spectrum, in order to search for differences that can be quantitatively characterized by terahertz spectroscopic methods, for the lowest energy transitions such as those that may be expected due to heating above the smoke point.

In the present work, we compare several off-the-shelf oils in the terahertz frequency range in raw state as well as after heating above their corresponding smoke points. The experimentation is performed using a fibre-coupled terahertz time-domain spectroscopy (THz-TDS) system in a transmission-mode set-up in the 0.05–2 THz frequency range that can potentially be conveniently used in the food industry.

The structure of this paper is organized as follows. First, the measurement system in the 0.05–2 THz frequency range and the sample holder are described. Then, the measurement process is outlined. Finally, the measured results are presented and conclusions are drawn.

## 2. Analysis

### 2.1. The terahertz system

The transmission spectrum is calculated from a time-domain acquisition of terahertz pulses through the sample. A reference spectrum is firstly obtained to characterize the system and the sample holder without the oil, and then repeated with the various oil samples.

The terahertz system consists of a 120 fs, 80 MHz mode-locked Ti:Sapphire laser (Mai-Tai II, Spectra Physics) operating near 800 nm, together with a T-Ray 2000 (Picometrix) analytical system. The Picometrix spectrometer is a THz-TDS system consisting of fibre-coupled transmitter and receiver heads. An oscillating mechanical translation stage adds a sinusoidally varying time delay to the receiver head, and provides a time-resolved measurement of the transmitted terahertz pulse. A Fourier transform of the acquired pulse results in the spectral response of the entire system. The spectrum of the terahertz radiation is measured with the sample in place and again in the absence of the sample. Subtracting the spectra of the system with and without the oil sample, gives the transmission spectrum of the sample alone.

A tight 12 mm collimated beam maximizes the available dynamic range of the system as well as maximizing the available system bandwidth. The data are acquired in rapid-scan mode that takes a few minutes to average 1000 samples. Each sample consists of 1024 points spanning a total time interval of 80 ps, with a fixed interval of 78.125 fs between consecutive data points. These constraints, together with approximately 55 *dB* dynamic range of the system, limit the spectral resolution to approximately 20 GHz.

### 2.2. The sample holder

**Figure 1.**
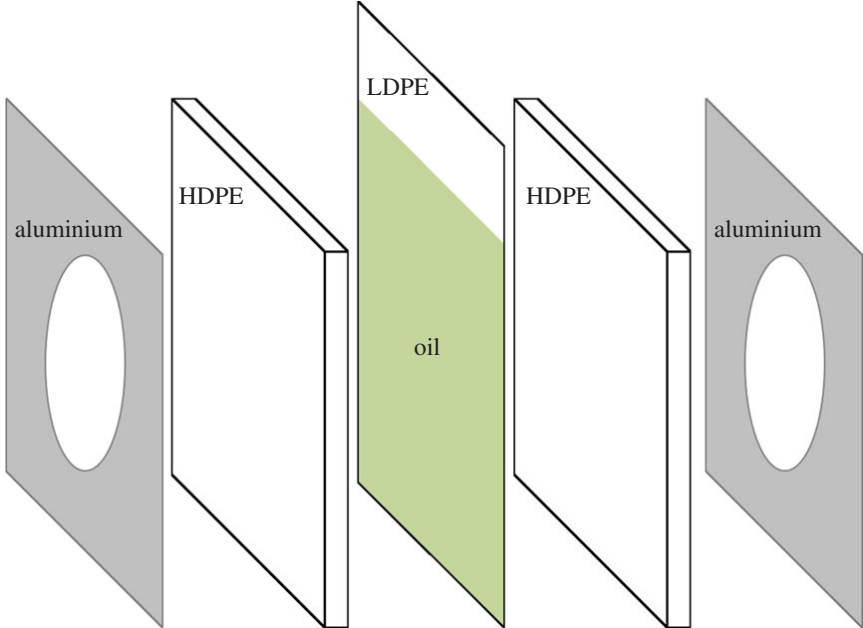
The sample holder structure. The oil sample is sealed in the LDPE bag that is pressed between the stiff HDPE sheets. The whole structure is pressed between the aluminium retaining mounts by screws and springs. The sample thickness is adjusted by a set of three mechanical micrometers (not shown in the figure). The empty upper part of the LDPE bag serves as a reservoir for the oil that remains outside the space between the two HDPE sheets.

To eliminate random errors due to alignment, the sample holder remains fixed during the course of the experiment. The oil under investigation is sealed within a 70 μ*m* thick low-density polyethylene (LDPE) material manufactured by Polybag Peak Packaging. This bag is then pressed between two 4.5 mm thick, stiff, high-density polyethylene (HDPE) plates that are supported by two aluminium retaining mounts with a 50 mm opening for the collimated terahertz beam, which is oversize compared to the 12 mm beam waist to avoid edge diffraction effects. The thickness of this sample holder sandwich structure is adjusted by a set of three micrometers placed uniformly around the 50 mm opening of each aluminium mount. These micrometer screws are reset to zero with the empty bag, and then adjusted to the corresponding thickness for the oil sample. This ensures that the walls of the sample are parallel and the sample is of correct thickness. Both HDPE and LDPE are transparent to the terahertz radiation [27,28], and are practical materials for this application. The sandwich structure of the sample holder is shown in figure 1. It is similar to the one suggested in [26], with improved controllability of the sample thickness. The THz-TDS system operates at room temperature in ambient air, and the reference terahertz signal is acquired with two LDPE sheets pressed between the stiff HDPE plates, thus serving as an empty LDPE bag or a sample with a zero thickness.

### 2.3. Measurement methods

**Figure 2.**
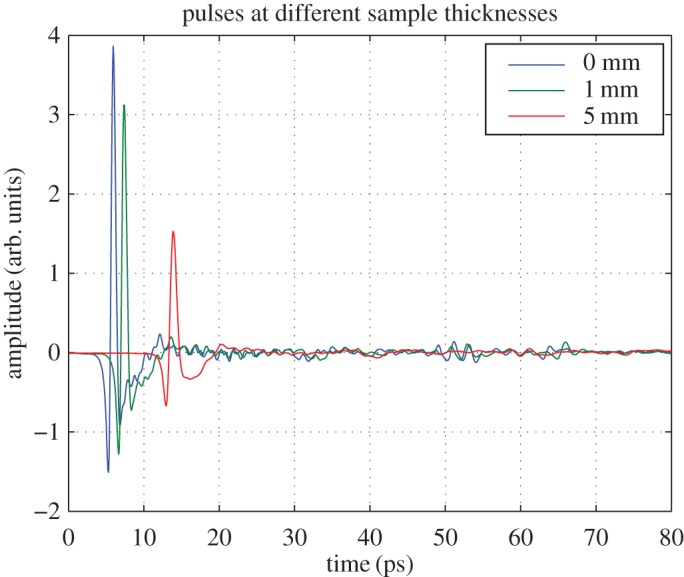
Reference pulse in the THz-TDS system through 0, 1 and 5 mm thick samples, using the sample holder described in figure 1. For the 0 mm case, the T-rays pass through two sheets of 4.5 mm HDPE and an empty 70 μ*m* LDPE bag. For the other measurements, the sample holder is adjusted to the corresponding thickness with raw refined olive oil inside the bag. For a given thickness, with the very similar refractive index of all the different oils, the Fabry–Pérot spectra will be essentially the same. Therefore, we have used this reference for all the acquired spectra.

The sample thickness is adjusted by a set of three micrometers, and each measurement is obtained by averaging 1000 consecutive scans for minimizing the noise fluctuations. The optical delay is set in such a position that the reference terahertz pulse has a peak at around 5 ps after the beginning of the 80 ps measurement window as shown in figure 2. The measured terahertz reference and sample signals are further windowed using a Blackman window function. The beginning of the measured data is zero padded and its end is correspondingly truncated, so that the medians of the peaks of the two terahertz pulses are in the middle of the windowing function. This ensures multiplication of the two pulse peaks by the same scaling factor.

The corresponding complex spectra of the two pulses, *H*_sam_(*ω*) and *H*_ref_(*ω*), are obtained through the usual Fourier transform technique. Then, dividing *H*_sam_(*ω*) by *H*_ref_(*ω*), the complex transfer function *T*(*ω*) is obtained as [29]

2.1 $$T(\omega )=\frac{H_{\mathrm{sam}}(\omega )}{H_{\mathrm{ref}}(\omega )}=\frac{4n(\omega )}{{\left[n(\omega )+1\right]}^{2}}e^{-j[n(\omega )-1-j\kappa (\omega )](\omega d/c)},$$

where *n*(*ω*) is the frequency-dependent refractive index, *κ*(*ω*) is the frequency-dependent extinction coefficient, *ω* is the angular frequency, *d* is the sample thickness and *c* is the speed of light in a vacuum. Then, from equation (2.1) the corresponding optical parameters—the refractive index *n*(*ω*) and the extinction coefficient *κ*(*ω*)—are obtained through the familiar expressions [29]

2.2 $$n(\omega )=1-\frac{c}{\omega d}∠\dot{T}(\omega )$$

and

2.3 $$\kappa (\omega )=\frac{c}{\omega d}\left\{\ln\left[\frac{4n(\omega )}{{\left[n(\omega )+1\right]}^{2}}\right]-\ln|\dot{T}(\omega )|\right\}.$$

From equation (2.3), the frequency-dependent absorption coefficient is also derived:

2.4 $$\alpha (\omega )=2\frac{\omega \kappa (\omega )}{c}.$$

In THz-TDS, special interest is paid to the refractive index and the absorption coefficient spectra of the investigated substance. Those parameters are derived from the measurement data through numerical implementation of the corresponding expressions (2.2) and (2.4).

## 3. Discussion and results

Previous studies show that the spectra of the different edible oils are very similar in the near-infrared spectral range [11]. As we shall find, this is confirmed in this paper also for the 0.05–2 THz spectral range. The spectra at lowest frequencies are most adequately measured at the maximum sample thickness while the spectra at highest frequencies are most adequately measured at the minimum sample thickness. This is due to the fact that the optimal sample thickness is inversely proportional to the frequency [30].

Samples are prepared for six different off-the-shelf oils: coconut, canola (i.e. rapeseed), rice bran, extra virgin olive, sunflower and refined olive oil. The optical parameters are measured for the uncooked oils. After that, all the oils are heated for 5 min above their corresponding smoke points [31] as shown in table 1. We have selected this time period to correspond with a typical culinary frying scenario. Then, the oils are cooled down to room temperature and their optical parameters are measured again.

**Table 1.** Smoke point temperatures of the edible oils under investigation.

edible oil	coconut	canola	rice bran	olive extra virgin	sunflower	olive refined
smoke point	177^°^*C*	204^°^*C*	232^°^*C*	191^°^*C*	210^°^*C*	199^°^*C*

**Figure 3.**
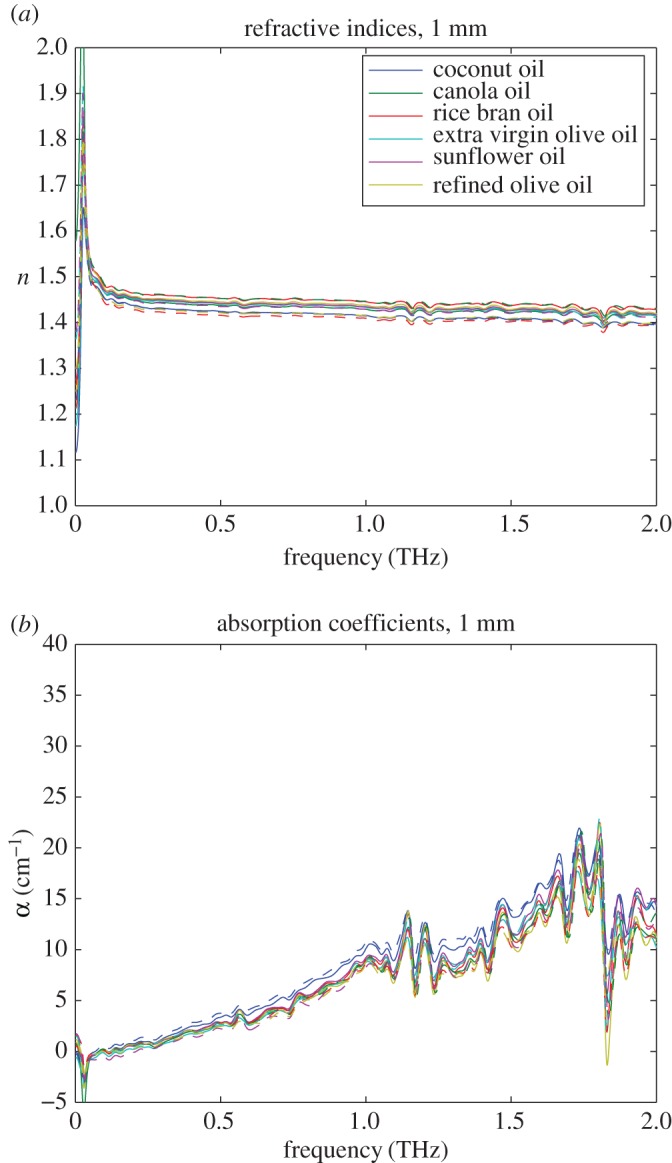
Measurement results for edible oil sample thickness 1 mm in a raw state (dashed lines) and after heating above their smoke point (solid lines). The similarity in the spectra is evident. The 1 mm sample thickness optimizes signals above 1 THz. Error bars above 1.8 THz become significant compared to the size of the spectral features. Errors also increase below 0.5 THz due to the low absorption, and the artefact at the low frequency is due to this effect. (*a*) Refractive indices for 1 mm samples and (*b*) absorption coefficients for 1 mm samples.

**Figure 4.**
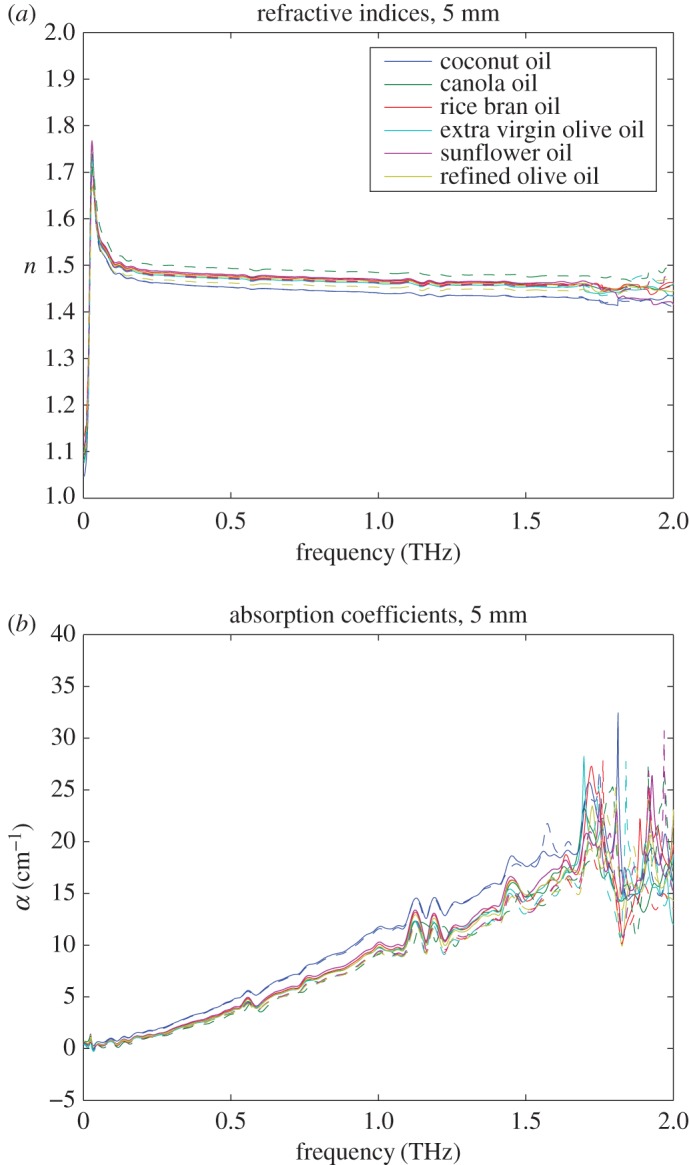
Measurement results for edible oil samples thickness 5 mm in a raw state (dashed lines) and after heating above their smoke point (solid lines). The similarity in the spectra is evident. This measurement optimizes signals below 1 THz. Above 1.5 THz, the errors become significant relative to the size of the spectral features, and above 1.7 THz, the absorption of the terahertz wave in the thick sample is such that the signal-to-noise ratio drops below the noise floor. (*a*) Refractive indices for 5 mm samples and (*b*) absorption coefficients for 5 mm samples.

We performed measurements at the appropriate oil thicknesses to extract the maximum dynamic range and high-frequency data from the measurements. When using a sample of 1 mm thickness, the highest frequency with reliable measurement is above 2 THz as shown in figure 3. For a sample of 5 mm thickness, the highest frequency with reliable measurement is about 1.7 THz as shown in figure 4. This is due to the fact that the thicker sample increases the linear dispersion of the pulse, and decreases the measurable bandwidth. On the other hand, the measured spectra for the sample with 1 mm thickness show significant fringes due to Fabry–Pérot reflections [29]. Also, for a sample of 1 mm thickness, a negative absorption coefficient is measured at the lowest frequencies. This is an artefact associated with the high level of measurement uncertainty at the lowest frequencies—there is simply not enough substance in the optical path to interact with the terahertz beam for producing meaningful results at this frequency and the signal-to-noise ratio of the terahertz pulse is below the noise floor. In [30], the authors derive the optimal thickness of a given test sample to be 2/*α*, for minimizing the measurement uncertainty. Indeed, it can be easily seen by visual inspection of the plots in figures 3 and 4 that for the frequencies around 0.5 THz the optimal thickness is about 2/4 cm=5 mm and for the frequencies around 2 THz the optimal thickness is about 2/20 cm=1 mm.

It is seen that all the different oils have very similar spectra and similar resulting optical parameters in the 0.05–2 THz frequency range. The strong spectral features common to all of the oils above 1 THz are probably due to the underlying chemistry common to all of the oils. Clearly, heating the oils above the smoke point only causes slight changes in the structure of the spectra and the resulting optical parameters in that frequency range. A previous study of the 0.2–1.5 THz spectra of raw versus pre-heated corn oil [5], also found slight differences. Our results confirm this and extend it to coconut, canola, rice bran, various olive and sunflower oils, with spectra measured out to more than 2 THz. Heating the oils above their corresponding smoke points and cooling them down to room temperature for a comparison of terahertz measurements has not been previously studied. We find that the measured slight differences are within the measurement uncertainty limits [30] and thus do not confirm changes in the spectra in the 0.05–2 THz frequency range.

## 4. Conclusion

This paper presents a THz-TDS set-up for the transmission mode inspection of edible oils in the food industry. Measurement results are presented for common edible cooking oils in the 0.05–2 THz range, before and after a 5 min heat treatment at the smoke point. Over this frequency range, no major changes occur in the spectra of the edible oils. The only minor change is a slight increase of the absorption coefficient of the oils that have been heated above the smoke point but this slight change is within the measurement uncertainty. This result contrasts with [6] showing significant spectral differences with old swill oils that have undergone more extensive heat treatments in contact with other substances including foods.

In [11], the authors show significant unique absorption features of different oils around 21 THz and around 36 THz—therefore future studies on heat-treated oils may benefit from further exploration into frequencies above 2 THz. A further possibility is to exploit synchrotron radiation in the FIR range to investigate changes in absorption features due to heat treatment. Furthermore, the experiments for this study are performed without food in the heated edible oils. Additional research is needed for investigating the effect of food on molecular changes in the oil above the smoke point. The same set-up can be used after heating the edible oils with different food substances in them, cooling them down to room temperature, and then filtering them to remove food particles.

## Data accessibility

The datasets supporting this article have been uploaded as the electronic supplementary material. http://dx.doi.org/10.13140/RG.2.2.35567.02729.
